# Prevalence of Perinatal Depression and Its Associated Risk Factors among Nepalese Women in Kathmandu, Nepal

**DOI:** 10.3390/healthcare12171773

**Published:** 2024-09-05

**Authors:** Pratikshya Wasti, Prem Prasad Panta, Vijay S. Gc, Biwash Ghimire, Pooja Sapkota, Sharada Prasad Wasti

**Affiliations:** 1 Department of Public Health, Nobel College, Kathmandu 44600, Nepal; 2 KIST Medical College and Teaching Hospital, Lalitpur 44700, Nepal; 3 School of Human and Health Sciences, University of Huddersfield, Huddersfield HD1 3DH, UK; vijay.gc@hud.ac.uk; 4 College of Pharmacy, Idaho State University, Pocatello, ID 83209, USA; biwashghimire@isu.edu (B.G.); poojasapkota@isu.edu (P.S.); 5 School of Human Sciences, University of Greenwich, London SE10 9LS, UK

**Keywords:** pregnancy, postpartum, perinatal depression, cross-sectional survey, Nepal

## Abstract

Perinatal mental health is a major public health issue that arises during pregnancy and/or after birth, with substantial implications for social, parental, and maternal functioning, as well as overall quality of life. The study aimed to determine the prevalence of perinatal depression and its associated risk factors among women who visited a maternity hospital in Kathmandu, Nepal. A cross-sectional study was conducted at the Paropakar Maternity and Women’s Hospital in Kathmandu. A total of 300 women in their perinatal period were interviewed. The Edinburgh Perinatal Depression Scale (EPDS) was used to measure perinatal depression. The Poisson regression model was used to determine risk factors associated with perinatal depression. The mean age of respondents was 25.5 (SD 4.5) years; average age during their first pregnancy was 23.5 (SD 3.7) years; and 53.7% of respondents were in the antenatal period. The prevalence of depressive symptoms (EPDS ≥ 10) was 40% (95% CI 31.4% to 45.8%). Unsupportive family members (adjusted prevalence ratio [aPR] 2.23; 95% CI 1.75–2.86), postnatal period (aPR 2.64; 95% CI 1.97–3.53), complications faced during delivery (aPR 1.76; 95%CI 1.30–2.39), history of intimate partner violence (aPR 0.48; 95% CI 0.36–0.64), and first pregnancy at the age of ≤25 years (aPR 0.61; 95% CI 0.42–0.88) were identified as key risk factors of perinatal depression. Strong family support and the active involvement of partners in counselling can contribute to alleviating perinatal depression symptoms. Targeted interventions in health and well-being services should be implemented to address mental health burden during both pregnancy and postpartum periods.

## 1. Introduction

Mental health is a global health issue. The importance of mental health is highlighted in the global literature, which emphasises the notion that there is “no health without mental health” [1], “no wealth without mental health” [2], and “no health without perinatal mental health” [3]. Perinatal mental health is also a significant public health concern that arises during pregnancy and/or within the postnatal period. Research indicates that up to 25% of women may experience perinatal depression [4], and the condition is more prevalent in low- and middle-income countries (LMICs), where there is a dearth of mental health care providers, resulting in a high rate of undiagnosed perinatal depression [5,6]. Perinatal depression has been linked to adverse effects in infants and children, including impaired cognitive, emotional, behavioural, and physical function. It considerably affects social functioning, parental and maternal functioning, and overall quality of life [7,8]. The treatment of perinatal depression in the early stages of pregnancy is critical because persistence leads to low mood, slow thinking, reduced physical activity [9], and increased risk of both physical and mental health issues [3,10]. These health issues have an impact not only on maternal health and pregnancy outcomes (i.e., low birth weight, preterm birth, and decreased movement and heart rate of the foetus), but also on the child’s short- and long-term developmental pathways [11,12]. Evidence shows that depressed pregnant women often decline treatment due to concerns regarding their foetus’s health [13]. Therefore, it is crucial to increase access to perinatal mental health services at an early stage for pregnant women and continue follow-up until the postpartum period.

The prevalence of perinatal depression varies depending on measurement disparities and cultural backgrounds, with the World Health Organization (WHO) reporting rates ranging from 12 to 42% in LMICs and 3.5% to 63.3% in Asian and Middle Eastern countries [14,15]. However, antenatal depression rates also vary between 4% and 46.8% [16] depending on the trimester of pregnancy, respondents’ socio-cultural backgrounds, and measurement disparities. Similarly, previous studies conducted in Nepal have also reported a highly varied prevalence of perinatal depression, ranging from 24.8% in a community-based study in Lalitpur District [17] to 41.5% in a district in Madesh Province [18]. Perinatal depression is more likely to occur in the presence of certain factors, which may be physical, obstetrical, familial, or economic. Several factors that influence both the mother and the child are associated with this condition, including impaired mother–infant relationships and the mental development of infants [4,19,20,21]. The socio-cultural circumstances in Nepal worsen the intricacies related to perinatal mental health burdens. Stigma, gender inequality, deeply ingrained myths, and false beliefs about mental health, including poorly detected and treated perinatal mental health, all have a significant impact on women’s experiences and access to appropriate care and support during the perinatal period [17,22,23]. 

Given the various risks women face during the perinatal period, it is equally crucial to find effective targeted intervention programmes to improve their health and quality of life. Noorbala et al. reported that psychiatric interventions could increase mental health services for perinatal women and address these burdens [24]. However, the evidence linking perinatal depression and potential risk factors in Nepal is inconclusive. Therefore, this study aimed to determine the prevalence of perinatal depression and its associated risk factors among women who visited a maternity hospital in Kathmandu. The findings will enable policymakers and stakeholders to develop practical and evidence-based interventions to reduce the prevalence of perinatal depression in Nepal.

## 2. Methods and Materials 

### 2.1. Study Setting and Populations

An analytical cross-sectional study was conducted at Paropakar Maternity and Women’s Hospital, which is one of the tertiary referral maternity hospitals in Kathmandu, Nepal, during May and June 2023. The survey was conducted with women aged 18 and older during their perinatal period (pregnancy and postpartum) who visited the hospital for maternity care but did not have any psychiatric problems. The primary reason behind selecting this hospital was that it is one of the largest (415-bed) and centrally located referral public hospitals in Kathmandu, where over 19,000 women give birth annually [25]. The perinatal period is defined as the period from gestation to one year after birth for women who come for antenatal, delivery, postnatal, or immunisation care in a hospital. Women who did not consent to participate and those with sick children were excluded from the study.

### 2.2. Sample Size and Sampling

A total of 300 perinatal women were recruited for this survey, and all respondents seeking maternity services were approached and interviewed using hospital outpatient and inpatient records. 

The sample size was calculated using the following formula:

$$n=\frac{Z^{2}pq}{d^{2}}$$

where Z = 1.96 at a 95% confidence interval;

p = 0.25 [sample proportion] according to a cross-sectional study conducted on the prevalence and risk factors of perinatal depression among women in Lalitpur, Nepal [17];

q = 0.75 [1 − p]; 

d = 5% [permissible error].

So, $n=\frac{1.96^{2}\times 0.25\times 0.75}{0.05^{2}}$ = 286.

Applying a 5% non-response rate, the study’s sample size was determined to be 300.

The respondents of this study were women who visited Paropakar Maternity and Women’s Hospital in Kathmandu, either for antenatal checkups, deliveries, or postpartum care. Each woman was sampled and selected for the interview after consulting with the maternity ward nursing staff and reviewing hospital records of maternity services, such as antenatal care (ANC), immunisation, and postnatal care (PNC) services.

### 2.3. Data Collection Tools

A structured questionnaire was used to collect data on sociodemographic characteristics, perinatal depression, and potential risk factors. This study’s depression assessment was carried out using the Nepali version of the Edinburgh Postnatal Depression Scale (EPDS) [26], a widely used questionnaire for detecting depression symptoms in perinatal women [22,26,27]. The EPDS tool is externally valid, with a sensitivity of 92%, specificity of 95.6%, positive predictive value of 77%, and negative predictive value of 99.3% [26]. The tool consists of a self-reporting 10-item scale questionnaire with scores ranging from 0 to 3, with a minimum of 0 and a maximum of 30 points, with a higher score indicating a higher level of depressive symptoms. Previous studies, including one from Nepal [28], have included similar criteria [22,27], i.e., an EPDS cut-off score of 10 to assess perinatal depression symptoms. The instrument’s reliability was calculated using Cronbach’s alpha, and the result was 0.730, which was within the acceptable range [29]. We also collected information on maternal health and sociodemographic traits. Eligible respondents who agreed to participate in the study were interviewed privately using face-to-face structured interviews. The interviews were conducted by local female interviewers and public health graduates who had prior experience and knowledge of quantitative data collection procedures in Nepal. 

### 2.4. Data Management and Analysis

The completed survey questionnaires were double-checked for errors, missing information, accuracy, and consistency before being coded, entered, and analysed into STATA version 18.5. The data were examined using descriptive statistics such as frequency tables, means, percentages, and standard deviations. Poisson regression models with robust variance were used to determine risk factors associated with perinatal depression. Previous research has indicated that measuring prevalence ratios is more precise for cross-sectional data with high prevalence rates and better accounts for confounders and interactions than logistic regression models [30,31,32]. The dependent variable was “depression symptoms”, and the independent variables were sociodemographic and maternal-health-related variables. Depression symptoms were coded as 0 = no or 1 = yes. Crude prevalence ratios (PRs) were first computed using Poisson regression. Significant risk variables (*p* ≤ 0.05) from the unadjusted analysis were included in the final model to estimate the adjusted prevalence ratio (aPR). The adjusted model included covariates such as age, ethnicity, religion, employment, and husband’s migration status.

### 2.5. Ethical Considerations

The study’s ethical approval was obtained from the Paropakar Maternity and Women’s Hospital (Reference No: 63/2046). Individual written informed consent was obtained from each participant, ensuring their anonymity and confidentiality.

## 3. Findings

### 3.1. Sociodemographic Characteristics of the Respondents 

A total of 300 perinatal women who completed the EPDS questionnaire were considered for analysis. The mean age of the respondents was 25.4 (SD 4.5) years, ranging from 18 to 42 years. Around two-thirds of respondents (62.4%) were from Janajati communities (ethnic minorities); more than three-quarters (77.3%) had completed higher secondary education and above; and more than two-thirds were unemployed (70.7%) at the time of the survey. Around half of the women (49%) had a nuclear family, with 51% having an extended family. Over three-quarters (76.7%) of the women received family support during the perinatal period. Almost all husbands (96.3%) had a formal education, and 97.0% were employed. Overall, 7.7% of respondents smoked, and 2.3% consumed alcoholic beverages during the perinatal period (Table 1).

Mothers’ average age during their first pregnancy was 23.5 (SD 3.7), ranging from 16 to 38 years. The number of respondents in the antenatal period was slightly higher (53.7%) than in the postnatal period (46.3%). Surprisingly, more than half of the respondents (55.3%) had unplanned pregnancies; the mean number of pregnancies was 1.5 (SD 0.7), and only 6.7% of respondents had had three or more pregnancies. Around one-fifth (15.7%, n = 47) of respondents had a history of miscarriage, whereas two women had aborted their pregnancy. Around three-fourths (73.4%) of women had a vaginal delivery, and 30.2% (n = 42) of respondents had complications during the delivery. High blood pressure (57.1%, n = 24) was the main complication, followed by preterm labour (21.4%) and heavy bleeding (14.3%) during the delivery. Over three-quarters (78.3%) of respondents received counselling services during the perinatal period. Above a quarter (26.7%) of respondents had a gender preference, and around two-thirds (62.5%) preferred sons over daughters.

Table 1 indicates that 120 respondents were found to be depressed based on the EPDS cut-off point of ≥10, indicating a prevalence of perinatal depression of 40% (95% CI 31.4% to 45.8%).

### 3.2. Unadjusted Risk Factor Analysis

An unadjusted prevalence ratio (uPR) was used to determine the impact of various factors on the likelihood of depressive symptoms (EDPS ≥ 10). Table 2 shows that perinatal depression was significantly higher in participants who identified as followers of religions other than Hindu (Buddhist, Christian, and Kirat) (uPR 1.52, 95% CI 1.13 to 2.04), were unemployed (uPR 2.21, 95% CI 1.45 to 3.37), had unsupportive families during the perinatal period (uPR 2.35, 95% CI 1.84 to 3.00) and postnatal period (uPR 2.50, 95% CI 1.83 to 3.41), and had complications during delivery (uPR 1.42, 95% CI 1.02 to 1.97). 

Similarly, not suffering from intimate partner violence (uPR 0.45, 95% CI 0.35 to 0.59), being 25 years of age or older (uPR 0.73, 95% CI 0.54 to 0.99), and receiving counselling services during the perinatal period (uPR 0.60, 95% CI 0.39 to 0.92) were identified as protective factors for perinatal depression symptoms. Respondents’ mean age, husband’s age, caste or ethnicity, being a left-behind wife, gender preference, history of miscarriage, and planned or unexpected pregnancy were not found to be significant predictors in the unadjusted analysis (Table 2).

### 3.3. Adjusted Risk Factor Analysis

After including all relevant factors (covariates) in the adjusted models, the risk estimate was slightly lower than in the unadjusted model. The adjusted prevalence ratio of depression was more than twice as high in women with unsupportive families (aPR 2.23, 95% CI 1.75 to 2.86) compared to those who received family support during their perinatal period. Women in the postnatal period had 2.64 times greater prevalence than women in the antenatal period (aPR 2.64, 95% CI 1.97 to 5.53). Similarly, women with a history of delivery complications had a 1.76 times (95% CI 1.30 to 2.39) greater prevalence of depression than those who did not. Moreover, the adjusted prevalence ratio showed that participants with no history of intimate partner violence (aPR 0.48, 95% CI 0.36 to 0.64) and those experiencing their first pregnancy at the age of 26 years or older (aPR 0.61, 95% CI 0.42 to 0.88) had a lower prevalence of depressive symptoms compared to their counterparts. However, the prevalence ratio between perinatal depression and receiving counselling services during the perinatal period did not vary significantly in the adjusted prevalence ratio analysis (Table 3).

## 4. Discussion

### 4.1. Prevalence of Perinatal Depression

The main finding of our study was the high prevalence of perinatal depression (40%) among women attending the hospital for maternity care, which was higher than previous studies conducted in Nepal. For example, a hospital-based study in Kathmandu reported a prevalence of 30.3% [22], 33.7% was reported at a regional hospital [33], 39% was reported in western parts of Nepal [23], and 41.5% was reported in a district in Madesh Province [18], determined using various measurement tools. Several studies in LMICs have reported comparable or even higher rates of perinatal depressive symptoms, such as 37% in Pakistan [34], 39.4% in Bangladesh [35], 42.8% in Afghanistan [36], and 46.8% in Thailand [16]. A recent systematic review and meta-analysis, however, reported a lower pooled prevalence (25.5%, 95% CI: 23.8–27.1) of perinatal depression in LMICs [9]. Likewise, previous studies conducted in Nepal that used an EPDS cut-off score of 10 or higher had rates ranging from 30.3% to 39.8% [22,23], which is slightly lower than our study. 

The reasons behind these highly varied findings could be attributed to geographical variations, as well as different screening tools, assessment timelines, and sample characteristics, including but not limited to postpartum depression and the EPDS cut-off of 13 and above criteria. In contrast, our study used an EPDS cut-off score of 10 or higher to identify potential perinatal depression symptoms, recommending early screening and treatment. The current study found a slightly higher prevalence of perinatal depression than previous studies conducted in Nepal’s central referral and regional hospitals. These studies used an EPDS score of 13 or higher as a cut-off point and only included postpartum mothers. However, in our study, we recruited women in the perinatal period from the central referral maternity hospital and used a lower cut-off score (EPDS ≥ 10) than previous studies conducted in Nepal. The prevalence of perinatal depressive symptoms varies greatly depending on screening tools, the perinatal period, sample size, and methodological variations.

### 4.2. Factors Associated with Perinatal Depression

The adjusted results suggested that unsupportive families or a lack of family support may be associated with an increased prevalence of perinatal depression. Women who did not receive family assistance throughout the perinatal period had over a two-fold higher prevalence of depressive symptoms than women who received family support. This finding is consistent with previous research showing that depressive symptoms are more likely in people who lack social support and have a bad connection with their family or partner [37,38]. Social support is vital in maintaining mental wellness, especially in countries like Nepal, where traditional family structure plays a crucial role in individuals’ lives. The feeling of being respected and cared for during and after the pregnancy may be linked to the improved emotional and mental well-being of the mother. This exemplifies the need for specific treatments and community awareness-building programmes about mental health concerns, as well as the timely detection, avoidance, and management of such conditions [9]. Healthcare service providers can include family members in prenatal counselling to help them understand the need for perinatal care, as well as run awareness-raising campaigns for the early identification, prevention, and treatment of mental health concerns.

Similarly, across the perinatal period, the postnatal period had a higher risk of depression than the antenatal period. This finding is consistent with earlier studies showing that taking care of a new child adds additional stress to women, and that children born from depressed mothers are more likely to have depression during their own adolescence and motherhood [39]. Therefore, support programmes during the perinatal period are crucial for all women, especially for working women, as an initiative to avoid postpartum depression. Our findings show that complications during delivery were related to a higher risk of depressive symptoms compared to deliveries without complications. Complications during childbirth, such as emergency caesarean sections, preterm birth, and maternal or neonatal distress, have been linked to increased rates of postpartum depression. These complications can contribute to feelings of loss of control, trauma, and anxiety, which are risk factors for developing depressive symptoms after delivery. A previous study in Nepal also revealed that women who delivered their last child by caesarean section were two times more likely to have depressive symptoms [33]. The study also reported that postpartum depression symptoms might continue anywhere from a month to years. According to Putnick et al., one-quarter (25%) of postpartum mothers had depression for three years following the birth of their child [40]. This shows that perinatal depression has long-term complications, and an inability to respond in the early stages might worsen health conditions. As a result, healthcare providers should provide counselling, monitor mothers for indications of depression, and refer them to appropriate mental health support services to ensure early detection, counselling, and treatment.

We found that women who went through intimate partner violence during the perinatal period had a higher prevalence of depression. This is consistent with previous studies conducted in Nepal [33,41] and elsewhere, in which interpersonal violence during pregnancy was identified as a predictor of perinatal mental health [34,42]. The Nepal Demographic and Health Survey (2022) reported that 27% of married women have experienced intimate partner violence. This proportion was much higher in Lumbini (29%) and Madesh Province (46%) [43]. There might be several reasons for having intimate partner violence, and understanding the causal pathway for this association is complex and multifactorial. Previous studies have indicated that the female sex of a newborn is associated with a higher rate of intimate partner violence. This is not an exceptional case to Nepal, as parents prefer boys over girls in general [44,45]. Therefore, good partner support at this vulnerable period of a woman’s life may play a protective role against perinatal depression. Addressing societal norms about gender preference and offering support to mothers regardless of newborn gender are crucial for alleviating the incidence of perinatal depression. 

Likewise, we found that pregnant women younger than 25 years of age had a higher risk of perinatal depression than those 26 years of age and older. Previous research has also indicated that younger mothers are at an increased risk of depression [46], and a recent study in Nepal reported that more than one-third (33.3%) of teenage mothers had depressive symptoms [47]. Several factors might contribute to this finding, including that younger mothers may face the challenges of their own development in addition to taking care of a newborn. In addition, early motherhood is associated with a lower degree of education and income, all of which can contribute to the development of depressive symptoms [46]. Early screening and supportive care, especially from the family and spouse, should be provided for younger women during the perinatal period.

The prevalence of depressive symptoms was lower among women who received counselling services in the unadjusted analysis. However, this finding was not significant in the adjusted analysis. Despite the statistical inconsistency, this finding indicates that it is crucial for healthcare providers to integrate early detection, prevention, and treatment into mental health services to avoid unintentional future consequences for mothers and newborns. An intervention study reported that the provision of mental health counselling during the initial phases of pregnancy led to a 95% effectiveness in improving perinatal mental health outcomes [24]. Therefore, the screening programme should target all pregnant women and provide ongoing counselling over the period to treat depressive symptoms. An earlier study showed that one-quarter (25%) of postpartum mothers had depression for three years following the birth of their child [40]. Therefore, health providers can include family members in prenatal counselling to help them understand the need for perinatal care, as well as run awareness-raising campaigns for the early identification, prevention, and treatment of mental health concerns over the period. 

### 4.3. Strengths and Limitations of the Study

To our knowledge, this is the first study based in a maternity hospital in Nepal assessing depressive symptoms during the perinatal period. Another strength of our study is the use of the EPDS tool, which is a widely used and validated tool for assessing perinatal depressive symptoms, including in Nepal. All data were collected through face-to-face interviews by trained researchers. The Poisson regression model with robust variance was used to determine risk factors associated with perinatal depression. Research indicates that measuring prevalence ratios is more precise for cross-sectional data with high prevalence rates and better accounts for confounders and interactions than logistic regression models [30,31]. 

Our study has some limitations. First, the study used a cross-sectional design, limiting its ability to establish a causal relationship between influencing factors and depressive symptoms. Birth cohort studies would have been ideal for determining the causal relationship between potential risk factors during the perinatal period. Perinatal depressive symptoms were self-reported, which is highly susceptible to over or underreporting and may result in recall bias. Therefore, clinical interviews should also be conducted to corroborate self-reported responses. Although this study was conducted in a hospital, it did not include women who missed their planned appointments during the study period. In consideration, the incidence of perinatal depression symptoms may have been underestimated. As a result, conducting a mixed-methods study involving multiple hospitals is required to gain a comprehensive understanding of perinatal mental health burdens.

## 5. Conclusions

Our study reported a high prevalence of perinatal depression. Unsupportive family members, the postnatal period, complications during delivery, history of intimate partner violence, and younger age of the mother at their first pregnancy were identified as key risk factors for perinatal depression. These findings suggest that health facilities should include a mental health screening programme for pregnant women, along with promoting a healthy lifestyle and providing more family and social support.. The provision of counselling services at the community or health facility and the integration of mental health screenings and counselling would help address perinatal mental health issues. Culturally appropriate interventions should be considered in culturally diverse societies to target associated risk factors such as intimate partner violence and unsupportive families during the antenatal and postnatal periods. Therefore, perinatal mental health should be a top priority in the national public health agenda, and policies should be put in place to ensure universal health coverage and provide ongoing follow-up over the perinatal period to treat depression symptoms.

## Figures and Tables

**Table 1:** Sociodemographic characteristics of the respondents (N = 300).

Variables	Frequency	Percent
Age of respondents—Mean age (SD)	25.4 (4.5), ranging from 18 to 42 years	
Religion		
Hindu	248	82.7
Buddhist	47	15.6
Christian	5	1.7
Caste/ethnicity		
Janajati	187	62.4
Brahmin/Chhetri	72	24.0
Madhesi	25	8.3
Dalit	16	5.3
Education level		
Illiterate	5	1.7
Basic education (1–8)	63	21.0
Higher secondary	196	65.3
Bachelor and above	36	12.0
Employment status		
Employed	88	29.3
Unemployed	212	70.7
Type of family		
Extended	153	51.0
Nuclear	147	49.0
Smoking (Yes)	23	7.7
Alcohol consumption (Yes)	7	2.3
Age of husband, mean (SD)	28.3 (4.0), ranging 20–43 years	
Received family support during perinatal periods	230	76.7
Mother’s age at first pregnancy, mean (SD)	23.5 (3.7), ranging 16–38 years	
Perinatal period of respondents		
Antenatal period	161	53.7
Postnatal period	139	46.3
Pregnancy planned	134	44.7
Miscarriage during the previous pregnancies	47	15.7
Type of delivery (n = 139)		
Vaginal delivery	102	73.4
Caesarean section	37	26.6
Had complications during delivery (n = 139)	42	30.2
Complications during delivery (n = 42) *		
High blood pressure	24	57.1
Preterm labour	9	21.4
Heavy bleeding	6	14.3
Foetal distress	5	12.0
Prolapsed umbilical cord	3	7.1
Eclampsia/preeclampsia	1	2.4
Received counselling services during the perinatal period	235	78.3
Had gender preference	80	26.7
Preference for a boy child	50	62.5
Prevalence of depression	120	40.0 (95% CI 31.4% to 45.8%)
EPDS < 10 (no symptoms)	180	60.0
EPDS 10 to 12 (mild symptoms)	74	24.7
EPDS ≥ 13 (high)	46	15.3

* Multiple response.

**Table 2:** Unadjusted model of the association between sociodemographic and maternal-related characteristics and depression status.

Characteristics	Depression Presence, n (%)	Depression Absence n (%)	Unadjusted PR	95%CI		*p*-Value
Respondents’ age in years						
Age 25 and below	68 (41.7)	95 (58.3)	Ref.			
26 years and above	52 (38.0)	85 (62.0)	0.91	0.69	1.20	0.510
Husband’s age						
29 years and younger	83 (41.1)	119 (58.9)	Ref.			
30 years and above	37 (37.8)	61 (62.2)	0.92	0.68	1.24	0.585
Caste/ethnicity						
Brahmin/Chettri	32 (44.4)	40 (55.6)	Ref.			
Janajati	70 (37.4)	117 (62.6)	0.84	0.61	1.15	0.29
Madhesi	16 (64.0)	9 (36.0)	1.44	0.97	2.13	0.068
Dalit	2 (12.5)	14 (87.5)	0.28	0.07	1.06	0.060
Religion						
Hindu	91 (36.7)	157 (63.3)	Ref.			
Others *	23 (44.2)	29 (55.8)	1.52	1.13	2.04	0.005
Current employment status						
Employed	19 (21.6)	69 (78.4)	Ref.			
Unemployed	101 (47.6)	111 (52.4)	2.21	1.45	3.37	<0.001
Being a left-behind wife						
Yes	18 (34.6)	34 (65.4)	Ref.			
No	102 (41.1)	146 (58.9)	1.19	0.79	1.78	0.402
Faced intimate partner violence						
Yes	14 (82.4)	3 (17.6)	Ref.			
No	106 (37.5)	177 (62.5)	0.45	0.35	0.59	<0.001
Age at first pregnancy						
24 years and younger	81 (44.8)	100 (55.2)	Ref.			
25 years and above	39 (32.8)	80 (67.2)	0.73	0.54	0.99	0.045
Received counselling during the perinatal period						
Yes	103 (43.8)	132 (56.2)	Ref.			
No	17 (26.2)	48 (73.8)	0.60	0.39	0.92	0.020
Received family support during the perinatal period						
Yes	70 (30.4)	160 (69.6)	Ref.			
No	50 (71.4)	20 (28.6)	2.35	1.84	3.00	<0.001
Maternity period						
Antenatal period	38 (23.6)	123 (76.4)	Ref.			
Postnatal period	82 (59.0)	57 (41.0)	2.50	1.83	3.41	<0.001
Had gender preference						
No	94 (42.7)	126 (57.3)	Ref.			
Yes	26 (32.5)	54 (67.5)	0.76	0.54	1.08	0.127
Suffered a miscarriage						
No	106 (41.9)	147 (58.1)	Ref.			
Yes	14 (29.8)	33 (70.2)	0.71	0.45	1.13	0.149
Was your pregnancy planned?						
Yes	55 (41.0)	79 (59.0)	Ref.			
No	65 (39.2)	101 (60.8)	0.95	0.72	1.26	0.740
Had any complication during delivery						
No	99 (37.9)	162 (62.1)	Ref.			
Yes	21 (53.8)	18 (46.2)	1.42	1.02	1.97	0.037

* Buddhist, Christian, and Kirat.

**Table 3:** Unadjusted and adjusted models of the association between maternal and social characteristics and depression status.

Factors	Level	Unadjusted			Adjusted *		
		PR	CI	*p*-Value	PR	CI	*p*-Value
Received family support during the perinatal period	Yes	Ref.			Ref.		
	No	2.35	1.84–3.00	<0.001	2.23	1.75–2.86	<0.001
Maternity period	Antenatal	Ref.			Ref.		
	Postnatal	2.50	1.83–3.41	<0.001	2.64	1.97–3.53	<0.001
Received counselling during the perinatal period	Yes	Ref.			Ref.		
	No	0.60	0.39–0.92	0.020	0.67	0.43–1.02	0.063
Complications during delivery	No	Ref.			Ref.		
	Yes	1.42	1.02–1.97	0.037	1.76	1.30–2.39	<0.001
Faced intimate partner violence	Yes	Ref.			Ref.		
	No	0.45	0.35–0.59	<0.001	0.48	0.36–0.64	<0.001
Age at first pregnancy	≤25 years	Ref.			Ref.		
	>25 years	0.73	0.54–0.99	0.045	0.61	0.42–0.88	<0.001

* Adjusted for age, ethnicity, religion, employment, and husband’s migration status.

## Data Availability

Data are contained within the article.
